# Cross-sectional analysis of eating patterns and snacking in the US Feeding Infants and Toddlers Study 2008

**DOI:** 10.1017/S136898001700043X

**Published:** 2017-03-20

**Authors:** Denise M Deming, Kathleen C Reidy, Mary Kay Fox, Ronette R Briefel, Emma Jacquier, Alison L Eldridge

**Affiliations:** 1 Nestlé Nutrition Global R&D, Florham Park, NJ, USA; 2 Mathematica Policy Research, Washington, DC, USA; 3 Nestlé Research Center, Public Health Nutrition, Route du Jorat 57, PO Box 44, Vers-chez-les-Blanc, 1000 Lausanne-26, Switzerland

**Keywords:** Snacking, Eating patterns, Pre-school children, Infants

## Abstract

**Objective:**

To explore eating patterns and snacking among US infants, toddlers and pre-school children.

**Design:**

The Feeding Infants and Toddlers Study (FITS) 2008 was a cross-sectional national survey of children aged 6–47 months, weighted to reflect US age and racial/ethnic distributions. Dietary data were collected using one multiple-pass 24h recall. Eating occasions were categorized as meals, snacks or other (comprised of all feedings of breast milk and/or infant formula). The percentage of children consuming meals and snacks and their contribution to total energy, the number of snacks consumed per day, energy and nutrients coming from snacks and the most commonly consumed snacks were evaluated by age.

**Setting:**

A national sample of US infants, toddlers and pre-school children.

**Subjects:**

A total of 2891 children in five age groups: 6–8 months (*n* 249), 9–11 months (*n* 256), 12–23 months (*n* 925), 24–35 months (*n* 736) and 36–47 months (*n* 725).

**Results:**

Snacks were already consumed by 37 % of infants beginning at 6 months; by 12 months of age, nearly 95 % were consuming at least one snack per day. Snacks provided 25 % of daily energy from the age of 12 months. Approximately 40 % of toddlers and pre-school children consumed fruit and cow’s milk during snacks; about 25 % consumed 100 % fruit juice. Cookies were introduced early; by 24 months, 57 % consumed cookies or candy in a given day.

**Conclusions:**

Snacking is common, contributing significantly to daily energy and nutrient needs of toddlers and pre-school children. There is room for improvement, however, with many popular snacking choices contributing to excess sugar.

The first 2 years of life is a period of rapid transition from an all-milk diet to a varied diet comprised of foods and beverages consumed by the family. It is also a time when the child transitions from infant feedings throughout the day to a pattern of meals and snacks. The timely introduction of complementary foods is essential to fulfil the nutritional and developmental needs of a child and to facilitate the dietary changes occurring from 6 to 24 months of age^(^ ^1^ ^)^. The early years are also a time when parental practices, food preferences and dietary habits are established^(^ ^2^ ^–^ ^4^ ^)^. Thus, structure and routine are particularly important considerations for parents when feeding their children. Recommendations for complementary feeding through the age of 2 years from the American Academy of Pediatrics encourage parents to establish predictable routines for meals and snacks, typically allowing 2 to 3 h between eating occasions, resulting in five or six eating occasions per day (e.g. three meals and two or three snacks), and to avoid ‘grazing’ behaviours with snacks or liquids^(^ ^1^ ^)^.

Snacking prevalence and energy intake from snacks increased among US pre-school children (2–5 years of age) from 1977 to 1996^(^ ^5^ ^)^ and continued to rise to 2006^(^ ^6^ ^)^. Although more recent national survey data in the USA show a significant drop in discretionary energy from sugar-sweetened beverages among pre-school children^(^ ^7^ ^,^ ^8^ ^)^, overall increases in snacking and the types of foods consumed as snacks have raised concerns over the potential role of snacking in the rise of childhood obesity^(^ ^9^ ^)^. Data from the 2002 Feeding Infants and Toddlers Study (FITS) showed that children aged 4–23 months consumed about 15 to 25 % of their daily total energy intake (TEI) from snacks which typically included milk, cookies, crackers, chips and fruit drinks^(^ ^10^ ^)^. National survey data from 1997 through 2006 indicated that pre-school children aged 2–6 years consumed approximately 27 % of their daily energy from snacks, the sources of which were often sweets (sweetened beverages and desserts) and salty snacks (such as crackers, chips, popcorn and pretzels)^(^ ^6^ ^)^. By 2011–2012, foods and beverages consumed as snacks contributed about 30 % of daily TEI in children aged 2–5 years and were important sources of key nutrients including dietary fibre, vitamin C and Ca^(^ ^11^ ^)^. Thus regular snacking may help young children meet their nutritional needs, but may also contribute to excess energy intake from energy-dense foods and beverages typically consumed as snacks.

It is clear from previous research that snacking plays an important role in the diets of young children, but a detailed look at snacking in the context of total daily intake is still missing. Therefore, the purpose of the current research is to explore snacking patterns among US infants, toddlers and pre-school children who participated in the FITS 2008. First, we explore the prevalence of snacking by evaluating the percentage of infants and young children consuming snacks. Next, we examine meal and non-meal energy intakes to understand the relative contribution of each eating occasion to daily TEI and to see when the energy contributions from different meals and snacks stabilize by age. Finally, we look at the contribution of snacks to nutrient intakes, along with the foods and beverages frequently consumed as snacks by infants, toddlers and young children.

## Methods

### Study design and participants

The FITS 2008 was a cross-sectional dietary intake study of a national sample of US children living in the fifty states and the District of Columbia, designed to obtain information on the diets and feeding practices of infants, toddlers and pre-school children^(^ ^12^ ^)^. As reported previously^(^ ^12^ ^)^, the full study sample was composed of 3274 children from birth up to the age of 4 years. The sample included both males (52·8 (se 1·44) %) and females (47·2 (se 1·44) %). The predominant race/ethnicity was Non-Hispanic White (56·2 (se 1·22) %), followed by Hispanic (21·3 (se 1·15) %), Non-Hispanic Black (14·2 (se 0·86) %) and Other races/ethnicities (8·3 (se 0·91) %). In total, 29·7 (se 1·32) % of participants received benefits from the Special Supplemental Nutrition Program for Women, Infants, and Children.

All study instruments and protocols, including incentives and informed consent procedures, were reviewed and approved by Mathematica’s independent institutional review board, Public/Private Ventures (Philadelphia, PA, USA). The current analysis includes data from 2891 infants, toddlers and pre-school children divided into five age groups: infants aged 6–8·9 months and 9–11·9 months, toddlers aged 12–23·9 months, and pre-school children aged 24–35·9 months and 36–47·9 months.

### Dietary intake methods

A single 24h dietary recall was collected for each child during a telephone interview using the parent or caregiver as a proxy. A second 24 h recall was collected on a random sample of 701 parents or caregivers to estimate usual nutrient intakes, but these are not reported here. Certified dietary interviewers from the Nutrition Coordinating Center at the University of Minnesota used the Nutrition Data System for Research (NDS-R, 2008; Minneapolis, MN, USA) to perform the dietary interviews. The NDS-R includes a computer-assisted multiple-pass approach to collect data on all foods and beverages consumed from midnight through midnight on the previous day. Interviewer probes and study tools, such as portion estimation illustrations specifically adapted for infants and young children, were developed for the 2008 FITS^(^ ^12^ ^,^ ^13^ ^)^. These were designed to minimize respondent error and increase accuracy in estimation of food intakes.

During the 24 h dietary recall, mothers or primary caregivers were asked the time of day of each eating occasion and whether they considered it to be breakfast, lunch, dinner, snack or other eating occasions. Accordingly, we then categorized all reported snack occasions by time of day, arbitrarily designating them as morning snack if eaten between waking until midday or lunch, an afternoon snack if eaten between midday or lunch until 18.00 hours, and an evening snack if eaten from 18.00 hours or dinner until bedtime. The category of other eating occasions was largely comprised of breast milk and infant formula for infants. For toddlers, other eating occasions were mostly milk, juice or water, items that parents apparently did not consider either a meal or a snack. Even though parents may be trying to regulate eating times, especially among infants, infant feeding (breast milk or infant formula) is not yet a meal or snack in the same way that meals and snacks are commonly understood when foods of the family table are eaten with the family. For this reason, we reclassified all breast milk and infant formula consumption into the category of other eating occasions. This allowed us to evaluate and understand the specific foods and beverages consumed as snacks in children at different ages throughout the complementary feeding period and into young childhood.

### Food group classification

All foods and beverages reported in the 24 h dietary recall were assigned to food groups using the categorization scheme of major and minor food groups used in the FITS 2002^(^ ^14^ ^)^, updated and expanded to include any new foods or beverages reported in the FITS 2008^(^ ^15^ ^)^. Foods commonly consumed at each eating occasion were calculated by categorizing foods eaten at each eating occasion and estimating the percentages of those who consumed specific foods within the major and minor food categories. For some categories, minor food groups were used to more accurately describe foods eaten. For example, the major food category ‘Milks’ was further classified into minor categories including breast milk, infant formula and cow’s milk.

### Statistical analysis

All analyses used sample weights and adjustments for non-response at different stages and population coverage, so that results appropriately reflect US age and racial/ethnic distribution of children from birth to 4 years of age. ANOVA was performed using the statistical software package SAS version 9.4 and means, percentages and standard errors were calculated. The *t* test was used to compare daily energy intakes from meals and snacks across age categories. Standard errors and *t* tests were calculated using the statistical software package SUDAAN release 9 (2005). The Bonferroni adjustment was used to adjust the individual *t*-statistics for multiple comparisons.

Descriptive statistics (mean and standard errors) were reported by age group for the per capita percentage of TEI by eating occasion, and the prevalence and the per consumer energy intake of meals, snacks and other eating occasions. Additional descriptive statistics were calculated to show: (i) the percentage of infants, toddlers and young children consuming individual meals and the total number of eating occasions; (ii) the percentage of energy from individual and total eating occasions in a day; (iii) the mean daily intakes of macronutrients and micronutrients for each eating occasion by age group; and (iv) the foods and beverages most frequently consumed at eating occasions based on the percentage of children consuming.

## Results

### Percentage consuming meals and snacks

The percentages of children consuming meals and snacks were lowest in infancy and reached levels during toddlerhood that continued through the pre-school stage (Table 1). About 85 % of infants aged 6–8·9 months consumed at least one meal on a given day and by the age of 9 months and through the pre-school stage, virtually all children consumed meals. About 37 % of 6–8-month-olds, 72 % of 9–11-month-olds and 95 % of toddlers and pre-school children consumed some type of food or beverage during snacking occasions on a given day (other than breast milk or infant formula). The afternoon snack was the most frequently consumed snack time across all age groups. The prevalence of the category for other eating occasions was lower especially after 12 months as the prevalence of breast-feeding and infant formula consumption was reduced. However, parents and caregivers reported various caloric beverages (mostly milk and juice) as other eating occasions for approximately 25 % of 2-year-olds and about 19 % of 3-year-olds.

**Table 1:** Percentage of US children consuming meals and snacks according to age category; Feeding Infants and Toddlers Study 2008

	6–8·9 months (*n* 249)			9–11·9 months (*n* 256)			12–23·9 months (*n* 925)			24–35·9 months (*n* 736)			36–47·9 months (*n* 725)		
Meal occasion	*n*	%	se	*n*	%	se	*n*	%	se	*n*	%	se	*n*	%	se
Total meals*	208	84·6	3·3	247	97·6	1·1	922	99·8	0·1	734	99·8	0·2	722	99·8	0·1
Breakfast	153	57·3	5·4	222	87·1	3·0	898	97·6	0·6	725	97·9	1·3	708	97·8	0·7
Lunch	165	68·6	4·5	221	84·3	4·2	877	94·4	1·2	710	97·1	1·0	699	97·4	0·7
Dinner	180	75·9	3·9	233	93·1	1·8	903	97·3	0·8	719	97·1	1·1	709	97·7	0·7
Total snacks†	96	37·1	5·3	175	71·9	4·2	875	93·6	1·3	698	95·2	1·1	691	93·7	1·8
Morning snack	32	11·8	2·9	76	34·4	5·0	564	60·8	2·5	449	59·8	3·1	434	57·2	3·1
Afternoon snack	63	22·5	4·8	127	42·6	4·6	762	83·1	1·8	601	76·4	3·0	578	75·2	3·1
Evening snack	39	14·1	3·5	61	28·0	4·6	477	49·9	2·6	386	50·3	3·2	428	60·6	2·9
Other‡	247	99·1	0·7	245	95·8	1·6	395	41·3	2·6	197	25·4	2·6	131	18·9	2·5
Breast milk/infant formula only	247	99·1	0·7	241	91·8	2·8	150	14·3	1·6	12	1·2	0·5	2	0·1	0·0

* ‘Total meals’ represents consumption of one or more meals during the day; meals are classified as breakfast, lunch or dinner.

† ‘Total snacks’ represents consumption of one or more snacks consumed during the day; snacks are classified by morning, afternoon or evening snack periods. Morning snack=anything called a snack and reported before midday or lunch; afternoon snack=anything called a snack and reported from midday or lunch until 18.00 hours; evening snack=anything called a snack and eaten from 18.00 hours or dinner until bedtime.

‡ ’Other’ represents all reported breast milk and infant formula consumption as well as any eating occasions reported by the parents or caregivers as other eating occasions.

### Contribution of meals and snacks to daily energy intake

**Fig. 1:**
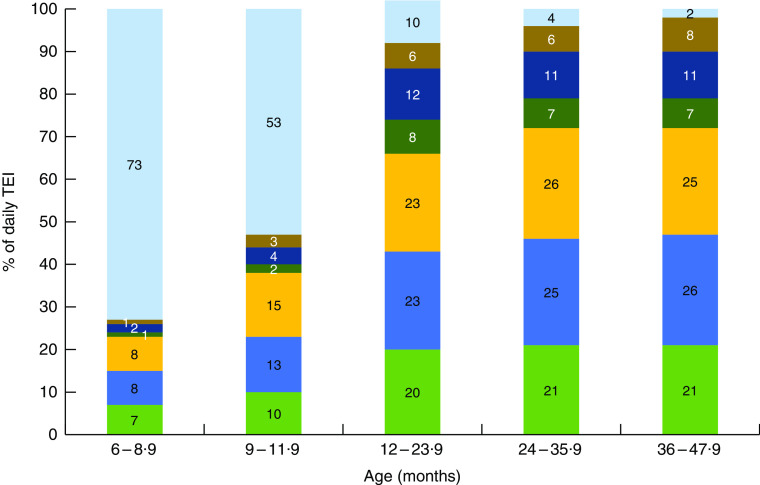
Percentage contribution of meals (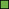, breakfast; 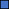, lunch; 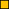, dinner), snacks (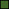, morning snack; 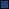, afternoon snack; 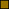, evening snack) and other eating occasions (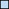) to total energy intake (TEI) of US children according to age category; Feeding Infants and Toddlers Study 2008

**Fig. 2:**
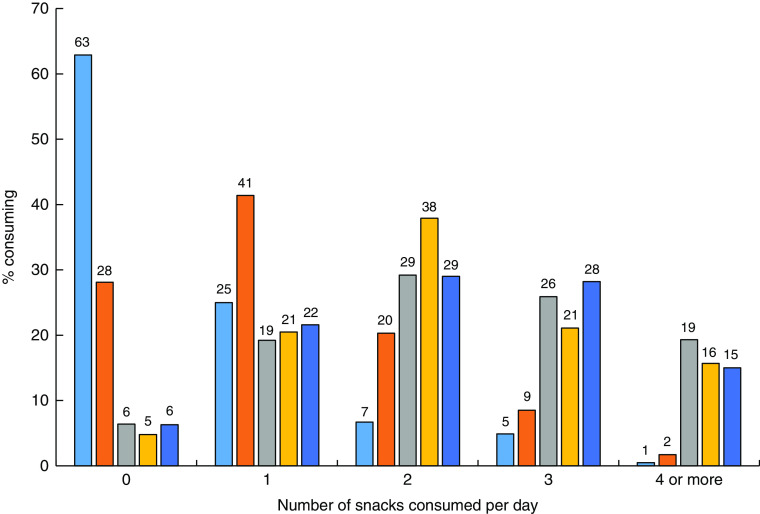
Number of snacks consumed daily by US children according to age category (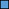, 6–8·9 months; 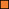, 9–11·9 months; 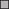, 12–23·9 months; 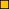, 24–35·9 months; 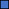, 36–47·9 months); Feeding Infants and Toddlers Study 2008

Figure 1 shows the changes in the percentage of TEI from meals, snacks and other eating occasions across age groups, illustrating which eating occasions become more or less important sources of energy in the diet at various ages during the transition from infant feeding to a pattern of meals and snacks. Breast milk and/or infant formula were the primary sources of energy for infants (Fig. 1), but beginning at the age of 6 months, meals and snacks became a more substantial portion of the diet comprising 27 % of TEI, with snacks contributing about 105 kJ/d (25 kcal/d; Table 2). Snacks contributed approximately 25 % of TEI for both toddlers and pre-school children, and by the age of 24 months, total daily snacks provided nearly as much energy as either lunch or dinner (Table 2). The contribution of morning and afternoon snacks to daily TEI appeared to stabilize at about 12 months of age, but evening snacks continued to increase in size through 47 months (Table 2). Most toddlers and pre-school children consumed two or three snacks daily (Fig. 2). Nearly 20 % of toddlers and about 15 % of pre-school children reportedly ate four or more snacks daily (Fig. 2).

**Table 2:** Total daily energy from meals and snacks among US children according to age category; Feeding Infants and Toddlers Study 2008

	Energy from food (kcal/d)*										
	6–8·9 months (*n* 249)		9–11·9 months(*n* 256)		12–23·9 months(*n* 925)		24–35·9 months(*n* 736)		36–47·9 months(*n* 725)		Across age categories
Meal occasion	Mean	se	Mean	se	Mean	se	Mean	se	Mean	se	*P* for trend†
Total day‡	726^d^	16	972^c^	39	1137^b^	18	1249^a^	24	1350^a^	24	0·0000
Total meals§	177^d^	16	394^c^	38	746^b^	16	896^a^	21	980^a^	21	0·0000
Breakfast	52^d^	8	105^c^	10	228^b^	7	258^a,b^	9	276^a^	9	0·0000
Lunch	62^d^	7	128^c^	13	256^b^	8	315^a^	10	354^a^	10	0·0000
Dinner	63^d^	6	161^c^	20	263^b^	8	323^a^	10	351^a^	12	0·0000
Total snacks║	26^c^	4	93^b^	15	288^a^	11	307^a^	12	340^a^	13	0·0000
Morning snack	6^b,c^	2	24^b^	5	93^a^	6	91^a^	8	86^a^	6	0·0000
Afternoon snack	14^b^	3	39^b^	9	132^a^	7	137^a^	8	148^a^	9	0·0000
Evening snack	6^d^	1	30^c,d,e^	9	63^b,c^	4	79^a,b^	8	106^a^	9	0·0000
Other¶	523^b^	19	484^b^	21	102^c^	9	46^d^	6	29^d^	5	0·0000
Breast milk/infant formula only	492^b^	16	454^b^	21	45^c^	7	2^d^	1	0·3^d^	0·2	0·0000

^a,b,c,d,e^Mean values across age categories within each eating occasion with unlike superscript letters were significantly different (*P*≤0·01). The Bonferroni adjustment was used for the individual *t*-statistics to correct for multiple comparisons.

* To convert to kJ/d, multiply kcal/d value by 4·184.

† Overall age comparisons are significant at the 99 % confidence level.

‡ ‘Total day’ represents all meal, snack and other eating occasions.

§ ‘Total meals’ represents consumption of one or more meals during the day; meals are classified as breakfast, lunch or dinner.

║ ‘Total snacks’ represents consumption of one or more snacks consumed during the day; snacks are classified by morning, afternoon or evening snack periods. Morning snack=anything called a snack and reported before midday or lunch; afternoon snack=anything called a snack and reported from midday or lunch until 18.00 hours; evening snack=anything called a snack and eaten from 18.00 hours or dinner until bedtime.

¶ ‘Other’ represents all reported breast milk and infant formula consumption as well as any eating occasions reported by the parents or caregivers as other eating occasions.

### Contribution of snacks to daily nutrient intakes

The contribution of snacks to daily nutrient intakes can be found in Table 3. The percentage of energy from snacks can be used as a standard from which the relative percentages of each nutrient can be evaluated. Snacks eaten by infants aged 6–8 months and 9–11 months contributed 3·2 and 9·1 % of daily energy, respectively. Proportionally more of this snack energy came from carbohydrates and less from protein and fats. Along with the higher proportion of energy from carbohydrates, foods and beverages classified as snacks also provided higher folate, vitamin C, dietary fibre and Fe relative to the energy they provided in the diets of infants. From the age of 12 months, the percentage of energy from carbohydrates eaten as snacks (28 %) was higher than the percentage of daily energy from snacks (25 %), and total and added sugar were also proportionally higher (33 and 36 %, respectively). Vitamin C was proportionally higher than energy from snacks among toddlers aged 12–23 months and children aged 24–35 months, but not for children 36–47 months old. Snacks contributed nearly 20 % of the daily Fe and nearly 25 % of the daily Ca and fibre intakes among toddlers and pre-school children.

**Table 3:** Percentage of daily energy and nutrient intakes coming from snacks among US children according to age category; Feeding Infants and Toddlers Study 2008

Percentage (%) of each nutrient	6–8·9 months (*n* 249)		9–11·9 months (*n* 256)		12–23·9 months (*n* 925)		24–35·9 months (*n* 736)		36–47·9 months (*n* 725)	
coming from snacks*	%	se	%	se	%	se	%	se	%	se
Daily energy from snacks	3·2	0·5	9·1	1·6	24·8	0·8	24·6	0·9	25·0	0·9
Protein	2·7	0·4	6·8	1·0	19·5	0·9	17·2	0·8	17·7	1·0
Fat	1·2	0·3	4·5	0·8	22·7	1·0	22·9	1·1	23·8	1·1
Saturated fat	1·1	0·3	3·5	0·7	23·0	1·2	23·2	1·2	24·5	1·2
Carbohydrate	4·8	0·9	12·4	2·0	27·9	0·9	28·2	1·0	28·2	1·0
Total sugar	3·9	1·0	10·8	2·2	31·8	1·2	33·2	1·3	32·8	1·2
Added sugar	5·2	1·9	13·7	3·0	35·0	1·6	35·6	2·1	38·5	1·6
Dietary fibre	7·5	1·2	15·3	2·7	22·0	1·0	24·3	1·1	25·5	1·2
Vitamin C	4·4	1·8	10·6	2·2	25·9	1·5	27·3	1·8	25·9	1·6
Folate	4·5	0·9	11·6	1·8	19·6	0·8	18·7	1·0	18·9	1·1
Vitamin B_12_	3·8	0·9	8·4	1·7	21·5	1·3	18·8	1·2	19·3	1·2
Ca	3·0	0·5	8·4	1·4	25·5	1·4	23·0	1·2	23·0	1·2
Vitamin D	1·0	0·4	3·7	1·0	23·5	1·6	20·8	1·6	21·2	1·5
Vitamin E	2·8	0·9	8·2	1·4	24·1	1·0	24·8	1·2	24·4	1·3
Fe	4·9	0·9	12·6	2·0	19·6	0·9	18·4	1·0	18·4	0·9
Zn	3·6	0·8	9·4	1·5	20·5	0·9	18·6	0·9	18·6	0·9
Na	3·4	0·7	9·2	1·6	19·2	0·9	17·9	1·1	17·4	1·0
K	3·7	0·6	10·1	1·8	23·8	1·1	22·9	0·9	22·9	1·1

* ‘Snacks’ represents consumption of one or more snacks consumed during the morning, afternoon or evening snack periods.

### Foods and beverages consumed as snacks

The foods and beverages most frequently consumed as snacks are shown in Table 4. Among infants aged 6–8 months, the most commonly consumed foods included infant cereals and 100 % fruit juice, each consumed by about 25 % of these infants. Fruits, including baby food fruits, were consumed as snacks by approximately 14 % of infants. Baby food fruits accounted for more than half of the consumption of fruits among 6–8-month-olds and about one-third of the fruit consumption for 9–11-month-olds. The most commonly consumed fruits were apples and bananas (data not shown). Grain-based snacks including crackers, baby cookies and breakfast cereals (e.g. ready-to-eat cereals and cooked cereals such as oatmeal) were consumed by 6–9 % of 6–8-month-old infants. The most commonly consumed foods and beverages for older infants 9–11 months of age included both infant and breakfast cereals, crackers, fruits, 100 % fruit juice and cookies.

**Table 4:** Foods most commonly consumed as snacks by US children according to age category; Feeding Infants and Toddlers Study 2008: FITS 2008

	6–8·9 months (*n* 249)			9–11·9 months (*n* 256)			12–23·9 months (*n* 925)			24–35·9 months (*n* 736)			36–47·9 months (*n* 725)		
Selected food group	% consuming	g/capita	se	% consuming	g/capita	se	% consuming	g/capita	se	% consuming	g/capita	se	% consuming	g/capita	se
Dairy															
Cow’s milk	1·8	4·1	3·0	4·6	14·5	7·1	45·9	131·0	10·0	38·4	95·8	9·0	40·7	93·4	7·8
Cheese	0·8	0·2	0·1	1·6	0·6	0·4	12·7	3·4	0·5	12·3	3·5	0·5	12·9	3·9	0·7
Yoghurt	5·5	4·9	2·5	5·5	3·8	1·2	11·3	10·1	2·0	12·8	16·4	2·6	12·2	14·9	2·9
Grain foods															
Cereal bars	0·0	0·0	0·0	0·5	0·1	0·0	0·7	0·1	0·0	0·0	0·0	0·0	0·0	0·0	0·0
Infant cereals	26·8	4·1	1·3	17·2	5·8	2·3	1·3	0·7	0·3	1·5	0·5	0·5	0·0	0·0	0·0
Breakfast cereals*	8·3	0·5	0·3	18·4	1·5	0·5	14·3	2·0	0·3	10·5	2·0	0·4	8·2	1·6	0·3
Crackers†	6·4	0·6	0·3	15·1	1·3	0·3	28·6	4·8	0·5	24·1	5·8	1·0	28·3	6·1	0·7
Fruit and 100 % juice															
Baby fruits	7·3	6·8	2·6	10·0	12·2	7·0	3·5	2·0	0·6	0·0	0·0	0·0	0·1	0·0	0·0
Fruits	6·4	3·4	1·5	27·8	24·5	7·9	37·2	36·8	4·8	46·3	54·9	5·0	42·9	50·0	4·4
100 % juice	24·3	22·1	8·3	18·6	23·1	5·6	31·7	58·9	5·9	25·7	45·4	5·2	19·1	41·2	5·9
Discretionary sweets & snacks															
Cookies, cakes, pies	8·7	1·2	0·5	17·5	2·4	0·8	29·2	8·1	1·4	36·8	9·0	0·9	33·1	11·2	1·2
Candy	0·0	0·0	0·0	0·7	0·1	0·0	14·5	3·3	0·5	19·8	4·5	0·7	23·9	5·4	0·6
Sweetened beverages‡	0·2	0·1	0·1	4·9	23·2	21·2	14·0	24·6	4·6	19·9	42·7	8·1	17·7	35·2	5·3
Ice cream/ frozen desserts	2·0	0·2	0·2	3·0	0·5	0·3	6·1	4·0	1·1	8·0	5·9	1·2	12·2	8·9	1·6
Salty snacks§	0·3	0·0	0·0	2·1	0·1	0·0	11·5	1·6	0·3	14·6	2·0	0·3	18·0	3·1	0·5

* Breakfast cereals included both ready-to-eat cereals and cooked (hot) cereals.

† Crackers included different types of crackers as well as pretzels and rice cakes.

‡ Sweetened beverages included carbonated and non-carbonated sweetened beverages.

§ Salty snacks included potato chips, popcorn, cheese curls/puffs, tortilla chips and other types of chips and salty snacks.

Cow’s milk and fruits were the most commonly consumed foods and beverages during snacks for toddlers and young children 12–47 months of age, with 37–46 % consuming these foods. By the age of 12 months, the types of fruits consumed switched away from baby food fruits, and included predominantly apples, bananas, grapes and citrus fruits (data not shown). Cookies, crackers and 100 % juice were consistently among the top five foods consumed as snacks for these ages. Other foods and beverages consumed as snacks included fruit drinks, cheese, yoghurt and breakfast cereal. The frequency of cheese and yoghurt consumption was relatively consistent at approximately 12 % in both the toddler and pre-schooler age groups. Candy and sweetened fruit drinks were consumed by approximately 20 % of children beginning at the age of 24 months. Consumption of salty snacks increased from 11·5 % for toddlers to 14·5 % for 24–35-month-olds and 18·0 % for 36–47-month-olds.

## Discussion

The present study documents the dietary transitions from multiple infant feedings to a traditional daily eating pattern of three meals and snacks, and shows that this pattern is quite well established by 12–23 months of age. Snacking occasions were designated by the parent or caregiver during the dietary interview and refer to the eating occasion rather than the consumption of specific types of foods or beverages, similar to what is done during the National Health and Nutrition Examination Survey (NHANES) dietary interviews^(^ ^11^ ^)^. By 6 months of age, complementary foods and beverages were introduced, consistent with the timing indicated in infant feeding guidelines^(^ ^1^ ^)^. From 6 to 8 months of age, foods and beverages other than breast milk or infant formula contributed 27 % of TEI, with only 3 % considered to be a snack. By the age of 12 months, meals and snacks contributed a higher proportion of the diet and by the age of 24 months the meal and snack patterns appeared to be fairly set. When breast milk and infant formula were excluded, we found that toddlers and pre-schoolers consumed a quarter of their daily energy from snacks (25 % of TEI), roughly equivalent to the energy contribution from lunch or dinner. However, toddlers and pre-schoolers generally consumed multiple snacks per day; so, on average, the energy contribution of each snack was less than a meal. The total energy from snacks in FITS 2008 is somewhat less than that reported among 2–5-year-olds (30 %) from NHANES 2011–2012, but is consistent overall with energy reported from snacks among 2–19-year-olds (26 %) and among the total population (24 %)^(^ ^11^ ^)^.

Multiple meals and snacks throughout the day are appropriate to meet the nutritional needs of growing children and to provide energy for their high levels of activity^(^ ^1^ ^)^. In general, children in FITS 2008 were routinely fed multiple meals and snacks during the day. Breakfast, lunch and dinner were eaten by most infants (68–76 % at age 6–8·9 months) and virtually all toddlers (94–98 %) and pre-schoolers (97–98 %), which is consistent with the cultural norm of three meals daily in the USA. The previous FITS (2002) reported that over 89 % of 9–24-month-old children consumed breakfast, lunch and dinner^(^ ^10^ ^)^. On average, infants aged 6–11·9 months consumed two or three meals and one snack, combined with three or four infant feedings, resulting in a total of about seven eating occasions in a day. By the age of 12 months, children ate five or six times daily, consisting of three meals and two or more snacks, eating patterns which are in line with complementary feeding recommendations^(^ ^1^ ^)^. The American Academy of Pediatrics guidelines also encourage parents to avoid ‘grazing’ behaviours with snacks or liquids^(^ ^1^ ^)^, so it is concerning that nearly 20 % of toddlers and 15 % of pre-schoolers consumed four or more snacks in a day. As children get older, snacking has been associated with poorer nutrient intakes^(^ ^16^ ^)^ and frequent snacking has been associated with higher energy intakes, as reported among 9–15-year-olds in the USA and 6–13-year-olds in Mexico^(^ ^17^ ^,^ ^18^ ^)^.

The foods and beverages first introduced to infants between meals are the same types of foods they consume early in the complementary feeding period: infant cereals, 100 % juice, baby food and regular fruits, and in later infancy, breakfast cereals and crackers, consistent with NHANES data showing sources of energy in infant diets^(^ ^19^ ^)^. Our data show that cow’s milk was the most frequently consumed beverage from 12 months and fruits were the most commonly consumed foods eaten as snacks across all ages. This, too, is reflected in NHANES data for infants and toddlers, which show that cow’s milk contributed 22·4 % of TEI for toddlers and fruits contributed 2·3 and 4·8 % of daily TEI for infants and toddlers, respectively^(^ ^19^ ^)^. Consumption of healthful foods is associated with availability and accessibility^(^ ^20^ ^)^, and with parental encouragement and modelling^(^ ^21^ ^)^. Clearly, many mothers or caregivers use snacks to provide healthful options such as milk, fruits and 100 % fruit juices to their children, although, to be consistent with American Academy of Pediatrics guidelines, 100 % fruit juice consumption should be limited to 4–6 fluid ounces (118–177 ml) daily for children aged 1–6 years^(^ ^1^ ^)^. Foods and beverages consumed as snacks made important contributions to energy but also to micronutrients and dietary fibre on a daily basis.

The snacking story is not consistently positive, however. Nearly 9 % of infants aged 6–8 months were fed cookies and this doubled by the time infants were 9–11 months old. By 12 months of age, nearly a third of children were consuming cookies or other sweet desserts, and by 24 months of age, nearly 20 % of children were also eating candy or sugar-sweetened beverages as a snack. These snacking choices contributed more total and added sugar relative to the percentage of daily energy contributed by snacks across all age groups. Comparing infants and pre-schoolers, the snacking choices became less healthful and snacks contributed more added sugar and fewer vitamins and minerals relative to energy. Other studies have found that the reasons for this may have more to do with reward or helping to manage behaviour than in providing nutrition or because the child is hungry^(^ ^22^ ^,^ ^23^ ^)^. While there is nothing inherently wrong with an occasional treat, the concern about frequent consumption of nutrient-poor or energy-dense foods and non-nutritive caloric beverages is that they displace foods that provide necessary nutrients^(^ ^16^ ^)^. There is also some evidence that consumption of nutrient-poor or energy-dense foods (including sugar-sweetened beverages, salty snacks, cakes and other sweets) at 2 years of age is associated with their consumption later in childhood^(^ ^24^ ^)^. Conversely, healthy dietary habits established early are also associated with healthy eating later in life^(^ ^3^ ^)^.

The present study has several limitations. The FITS 2008 was a cross-sectional survey; so, similar to other cross-sectional surveys, it was not possible to examine the influence of different snacking behaviours on long-term outcomes of diet quality or health. The cross-sectional view does, however, provide valuable insight into eating patterns, including the number of snacks and the proportion of energy consumed during different meals and snacks, the frequency of snacking, the nutrients contributed and the foods commonly consumed as snacks. The study was completed in 2008, so although it may not completely reflect snacking patterns or exact foods consumed at the present, this in-depth view will provide an important comparison point for future studies. Combining energy and nutrients from multiple snacking occasions per day may increase the perception of the relative importance of snacks in the diet in comparison to individual meals. Nevertheless, the foods and beverages consumed as snacks contribute significantly to overall daily energy and nutrient intakes. We found inconsistencies in the way different parents classified their child’s consumption of breast milk or infant formula; by reclassifying all breast milk and infant formula consumption into the other eating occasions category, we may have slightly underestimated the nutrient intakes at meals and snacks where breast milk or infant formula could have been used in preparation of other dishes, such as infant cereal. However, because the patterns described herein are consistent with other research, the effect of this is likely to be very small. Similarly, some foods and beverages other than breast milk or infant formula were described by parents as other eating occasions and would also result in a small underestimation of the contribution of energy and nutrients from snacks. Finally, sample sizes were small for infants when looking separately at morning, afternoon and evening snacks, so information on energy intakes by time of day in these groups should be interpreted with caution.

## Conclusions

The rapid dietary shift occurring in the first few years of life is a critical time to establish eating patterns that fulfil the nutritional needs of the growing child and provide a foundation for daily eating habits that promote optimal health. The FITS 2008 clearly shows that by the age of 12 months, snacking is integrated into the daily diet and makes important contributions to daily energy and nutrient needs of infants, toddlers and pre-school children. However, the study also identifies areas for improvement, especially in regard to total and added sugars. Improvements could be made in encouraging smaller portions of high-sugar products and lower-sugar choices, as total and added sugar stand out as disproportional to the energy contribution from snacks. On the other hand, we see more than 40 % of children aged 12 months or older consuming milk and fruits as snacks, and more than 25 % consuming crackers, which contribute positively to intakes of Ca, dietary fibre and whole grains (depending on the type of crackers selected). These insights can help in developing guidance for health-care providers to educate parents on healthy snacking for young children, showing that snacks, if selected with care, can be important contributors to the child’s nutrition for the day. In order to educate or develop interventions around snacking that is not motivated by nutrition and feeding, but by situation and behaviour, a deeper understanding of snacking behaviours is required, and it will be important to verify these findings in future studies of infants, toddlers and children. Future research may also include the impact of location on snack choices and nutritional quality, deeper understanding of how parents define snacking, as well as parental motivation when selecting and offering snacks to their young children.
